# Finding the best fit for improving reproducibility: reflections from the QUEST Center for Responsible Research

**DOI:** 10.1186/s13104-022-06108-x

**Published:** 2022-08-03

**Authors:** Natascha Drude, Lorena Martinez-Gamboa, Tamarinde Haven, Constance Holman, Martin Holst, Silke Kniffert, Sarah McCann, Torsten Rackoll, Robert Schulz, Sarah Weschke

**Affiliations:** 1 QUEST Center for Responsible Research, Berlin Institute of Health at Charité – Universitätsmedizin Berlin, Charitéplatz 1, 10117 Berlin, Germany; 2 Institute of Ethics, History and Philosophy of Medicine, Medizinische Hochschule Hannover, Carl-Neuberg-Str. 1, 30625 Hannover, Germany

**Keywords:** Reproducibility, Institutions, Behaviour change, Responsible research, Stakeholders, Research culture

## Abstract

Increasing the reproducibility and trustworthiness of biomedical research requires engaging stakeholders from all levels in an institutional setting. The QUEST Center for Responsible Research aims to develop and implement new approaches to improve the culture and practice of research, tailored to the needs of these stakeholders. Members of the QUEST Center organised a brainstorm to reflect on the challenges and new opportunities encountered in implementing different projects through QUEST and share the lessons that working groups have learned over the first five years. The authors informally surveyed and interviewed working groups where relevant and highlight common themes that have influenced the success of many projects, including top-down and bottom-up engagement, managing expectations, the availability of expertise, ensuring sustainability, and considering incentives. The commentary authors conclude by encouraging the research community to view initiatives that promote reproducibility not as a one-size-fits-all undertaking, but rather as an opportunity to unite stakeholders and customise drivers of cultural change.

## Introduction

Trustworthiness forms the bedrock of scientific research. However, reports of low reproducibility [1, 2] and research waste [3–5] have shaken trust in biomedical research. The scientific community has responded with initiatives to evaluate and increase value in biomedical research, including non-profit organisations [6], national peer-led consortia [7], and funding policies [8, 9]. Reforms to how research is published [10], assessed [11, 12], and funded [13, 14] have ensued.

The QUEST Center for Responsible Research [15] was founded in 2017 within the Berlin Institute of Health (BIH), which was integrated into Charité – Universitätsmedizin Berlin in 2021. QUEST is an institutional initiative to advance the uptake of transparent and reproducible research practices and facilitate change in the practice and culture of research. Our organisation provides education, services, and tools, and performs meta-research to identify opportunities to improve research practices and assess the effectiveness of interventions. The QUEST Center currently has over forty active projects coordinated by eleven project teams targeting many different levels, from institutional governance to individual researchers at all career stages. This multi-level approach to promoting responsible research at an institutional level entails engaging with expertise, behaviour, and attitudes of stakeholders from across the spectrum of science and translation. Similar institution-level approaches aimed at research improvement are employed at the University of Zurich and Stanford University, among others, but QUEST provides a unique perspective due to its size and scope.

While the mission of QUEST has already been described in detail [16], this commentary reflects on experiences, collected via brainstorming sessions and interviews with QUEST colleagues, in facilitating change in research practice and culture. Achieving change can be difficult and is often undermined by resistance to new practices or practical limitations related to time or personnel.

Recently, members of the QUEST Center organised a brainstorming session to reflect on the challenges and new opportunities encountered in implementing different projects that aim to promote responsible research, through QUEST and share the lessons that working groups have learned over our first five years. The authors informally surveyed and interviewed different project teams and noted common themes that may have influenced the success of many projects, including top-down and bottom-up engagement, managing expectations, the availability of expertise, ensuring sustainability, and considering incentives.

Many experiences of different project teams in our organization may be captured by the idea that research improvement is a continuously evolving process that requires flexible and tailored solutions for stakeholders (for similar reflections, see [17, 18]. We hope that other scientists, initiatives, and institutions can benefit from experiences gained at QUEST over our first five years of science improvement activities.

## Main Text

### Top-down and bottom-up engagement

Initiatives to improve research culture and practice usually involve both top-down and bottom-up elements and interests. For example, top-down initiatives may be initiated by institutional leadership redefining missions and values that later cascade into larger-scale behaviour change at other levels. Bottom-up changes may be initiated by individual researchers or research groups identifying barriers and focusing on improvements in practice or research culture in their direct environment. One example that employed both top-down and bottom-up elements is a recent project where institutional leadership introduced a self-developed quality management system for preclinical laboratory work, after realising that established industrial quality standards are not a good fit for academic research laboratories. Here, experienced QUEST scientists worked together with laboratory researchers to identify their needs and develop a new, modular quality system and several supporting tools that were piloted in one research department [19, 20]. The PREMIER (Predictiveness and Robustness through Modular Improvement of Experimental Research) project team is currently adapting the system for use by other research laboratories. Attempts to align simultaneous bottom-up and top-down initiatives and identify overlap in the interests of different stakeholder groups can be challenging [21], especially in the context of research improvement [17, 18]. The approach of the PREMIER team united a top-down desire for high-quality research and transparent documentation with bottom-up needs for efficiency and support in experimental work.

### Managing expectations

Managing expectations—both our own and those of stakeholders with whom we engage—has emerged as a challenge across several projects and underscores the necessity for transparent bilateral communication. In several projects, initial enthusiasm from researchers waned when it became apparent that there were significant time and complexities associated with implementing new, more robust research practices. Learning to clearly convey the benefits of initiatives, but also their limitations and the scope of investment necessary from research teams, has been an important lesson. Several different project teams recommend addressing expectations of all stakeholders at an early project stage. For example, a QUEST team providing infrastructure and training for lab-based researchers has learned the importance of communicating that tools such as electronic laboratory notebooks will benefit transparency and documentation of daily work, but will not solve overarching structural problems of management or oversight [22]. Misalignment of expectations can contribute to poor uptake or early rejection of new tools or methodologies.

These themes also extend to education and training opportunities. For example, members of QUEST are developing implementation-focused training, which helps unite participants’ ideals and expectations related to reproducible research practices with everyday research challenges [23]. In addition, members of the QUEST education team are continually adapting curriculum design and course descriptions to help potential participants align their research needs and career plans with training and education opportunities.

### Expertise

Many research improvement initiatives at QUEST have required a broad range of skills within our working groups to understand current culture and practices, assess needs, and design, implement, and evaluate the effectiveness of interventions. This requires continuous exchange, often beyond our direct institutional environment, for example, consulting policy or industry experts. Transparent and honest debate between all actors is vital for improving teamwork, building trust, and establishing new co-operations. Many teams have found that it is highly beneficial to exchange experiences within interdisciplinary groups and share resources and skillsets across projects wherever possible.

Another successful method has been to assemble a working group with a range of complementary skills and a common goal, while allowing the project aims to evolve according to the group’s interests and expertise. This method was taken with an international group of software engineers and biologists and has resulted in a pipeline of automated tools that assess preprints or papers, for practices like open data or trial registration. Public reports from this ScreenIT pipeline provide individualised feedback to help authors to make their manuscripts more transparent and reproducible [24].

It is crucial that teams honestly reflect if they lack necessary skills or expertise needed for optimal project realisation and seek support from collaborators. In an initiative to provide tools and services to preclinical researchers, a team working to support adoption of electronic laboratory notebooks recently collaborated with experts in implementation science, and discovered significant limitations to their original plans that might otherwise have gone unnoticed [22]. Establishing new collaborations with experts in behaviour change management would likely help several teams identify additional opportunities for improving research culture and practice.

It is easy when looking through the lens of research improvement to assume that all processes can and should be improved. However, assessing, respecting and valuing existing (institutional) expertise and acknowledging that current practices may be easily justified, are equally important to create a well-fitted success story.

### Sustainability

A common issue faced by projects at the QUEST Center involves the sustainability of our research improvement approaches. Most initiatives require both buy-in from target stakeholders and top-down policies to provide the resources required for long-term cultural change. Usually, influencing these policies requires clear evidence of benefit or change but defining and evaluating concrete measures of success, especially in the short and medium term, can be difficult. The QUEST Data Science team has created a semi-automated dashboard that monitors reporting of factors that promote reproducibility in publications from researchers at our university medical centre over time [25]. This helps to highlight areas for improvement and evaluate the impact of interventions and policy changes. The dashboard recently received support from institutional governance for long-term implementation.

However, other projects developing tools and guidelines are heavily reliant on third-party funding. Without institutional or other sustained buy-in, their impact may be limited. Individuals and organisations may be hesitant to rely on a service or tool with an uncertain future. This is the case for a federally funded project developing guidelines for the validation of potential new drug targets [26]. The project was successfully completed and guidelines published [27], yet the team currently lacks the resources for continuing dissemination among potential users. These challenges are shared by projects that develop, for example, automated screening tools or datasets requiring long-term digital infrastructure. Within traditional academic structures, funding is often limited to the development phase of tools or services, and it can be difficult to secure support for their continued operation and dissemination. Future strategies for promoting reproducibility should consider methods to align bottom-up momentum and proof of principle with identification of top-down resources that are critical for the long term. This may include fine-tuning strategies for building independent competency within the community, or eventual project “hand-over” to institutions or other parties. For example, the QUEST Open Data and Research Management team found that library services at our institution were a key ally for long-term management of strategies related to open access publishing.

### Incentives

As is increasingly recognised across academic biomedicine [28], a lack of incentivisation for research improvement activities plays a large role in resistance to changing research practices. Researchers at our institution have explicitly expressed hesitance to devote resources to robust research practices that are not commonly incentivised as part of academic career progression. Modifying incentive structures has proven complex. At QUEST, the Incentives and Indicators team is specifically concerned with developing and implementing new approaches of incentives in the organisational reward, funding and hiring system. Unlike in some of QUEST’s other initiatives, the main target group of their efforts are not primarily the researchers themselves, but the organisational research governance bodies in the different areas of research assessment. These stakeholder groups encompass, e.g., members of management, research, and administration. The goals of the MERIT (Mechanisms of Robust, Innovative and Translational Research) project include new methods for assessing performance that incorporate open research practices during recruitment of scientific personnel, projects for intramural funding and assessment of doctoral theses [29, 30]. In addition, there are individual or research group awards for research improvement activities (e.g., for sharing open data, reporting null results or involving patient and stakeholder engagement) that have helped to increase awareness and acknowledged additional effort for open and robust research practices among local researchers.

To help address incentivisation, the Incentives and Indicators group is also developing an open source web-based app that gives appointment committees access to expanded quality-oriented and open science performance assessment criteria to use in hiring and promotion committees, the MERIT App [29, 30]. Support from university leadership and access to the relevant decision-makers and meetings to build trust, assess needs, and understand existing processes has been crucial to the success of this project. Changing incentives on a broad scale will need input from funders, industry partners, publishers, and other stakeholders. QUEST is actively engaging with these stakeholder groups to harmonise and broaden the uptake of appropriate incentives for reproducible research.

## Outlook

**Fig. 1:**
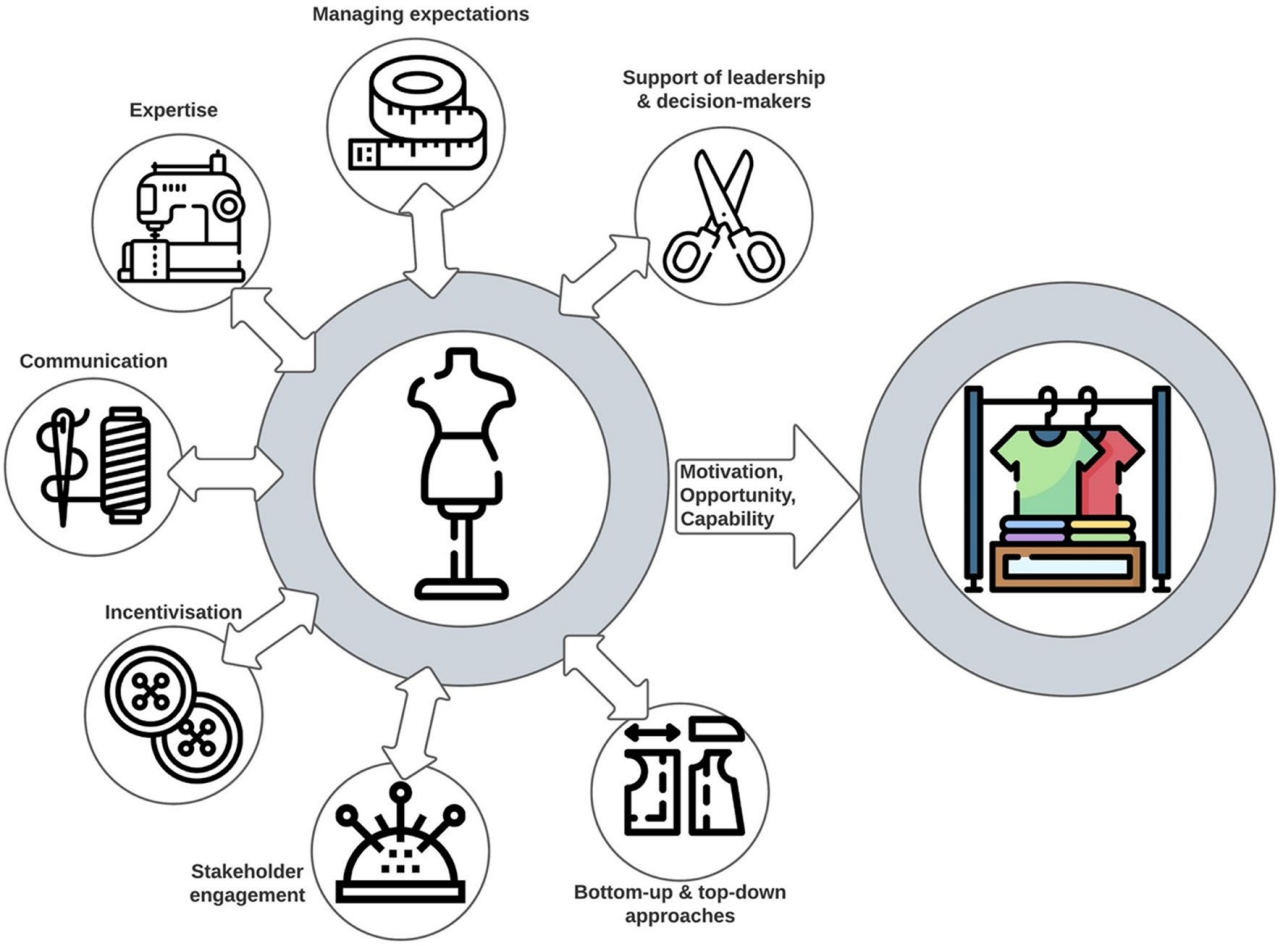
**Improving reproducibility requires a tailored approach.** There is no one-size-fits-all approach for improving the practice and culture of research. This schematic summarises key factors identified in QUEST Center projects that support creation of tailored results for stakeholders in reproducible research. These factors include support of key decision makers, managing expectations, employment of expertise, a successful communication strategy, identifying important incentives, stakeholder engagement, and united bottom-up and top-down approaches. Motivation, opportunity and capability, in a framework described by Michie et al. see also [16, 31] lead to a well-fitting initiative. Icons used in this figure adapted from resources on www.flaticon.com

Taken together, the experiences of many projects at the QUEST Center have driven home that improvement in the culture and practice of research is a process of continuous communication and adaptation, not a singular endpoint. This mindset can help support conversations around project sustainability and building long-term skills and trust within stakeholder communities. Many projects at the QUEST Center target different communities to implement new practices and facilitate institutional culture change in biomedical research. Consequently, their experiences have underlined the importance of continuously adapting research improvement initiatives and tailoring them to target groups. A project to improve the culture and practice of research often begins as a rough idea and requires many factors to become a final, well-fitting product, summarised in Fig. 1.

As a final note, our experiences at the QUEST Center have shown that research improvement activities can lead to new and unexpected directions, for example, collaborations with regulatory bodies and ethics boards in designing responsible preclinical research. It is important that organisations working to promote reproducibility acknowledge gaps in their expertise, be flexible, and remain open to new perspectives or approaches. This is particularly relevant for research improvement activities, where finding the perfect fit for cultural change is a challenging goal within an ever-changing landscape. There are many ways to advocate for increased reproducibility in research. In preparing this commentary, the QUEST writing group has noted that diverse projects often have shared challenges and keys to success. Critically reflecting on these elements has proved to be an enriching and informative experience for our organization.

## Data Availability

Not applicable.

## Abbreviations

BIH: Berlin Institute of Health
MERIT: Mechanisms of Robust, Innovative and Translational Research
PREMIER: Predictiveness and Robustness through Modular Improvement of Experimental Research
QUEST: Quality, ethics, open science, translation
